# In vivo-measured Lewy body pathology is associated with neuropsychiatric symptoms across the Alzheimer’s disease continuum

**DOI:** 10.21203/rs.3.rs-6270682/v1

**Published:** 2025-03-26

**Authors:** Douglas Leffa, Guilherme Povala, Pamela Ferreira, João Pedro Ferrari-Souza, Guilherme Bauer-Negrini, Matheus Rodrigues, Livia Amaral, Firoza Lussier, Marina Medeiros, Carolina Soares, Cristiano S. Aguzzoli, Arthur Macedo, Joseph Therriault, Pedro Rosa-Neto, Dana Tudorascu, Eduardo Zimmer, Bruna Bellaver, Tharick Pascoal

**Affiliations:** University of Pittsburgh; University of Pittsburgh; University of Pittsburgh; Brain Institute of Rio Grande do Sul; McGill Univeristy; Universidade Federal do Rio Grande do Sul; University of Pittsburgh; University of Pittsburgh

## Abstract

Intracellular alpha-synuclein aggregates, known as Lewy bodies (LB), are commonly observed in Alzheimer’s disease (AD) dementia. Post-mortem studies have shown a higher frequency of neuropsychiatric symptoms among individuals with AD and LB co-pathology. However, the effects of in vivo-measured LB pathology on neuropsychiatric symptoms in AD remain underexplored. This study aimed to evaluate cross-sectional and longitudinal effects of in vivo-measured LB pathology on neuropsychiatric symptoms across the AD continuum. We analyzed data from 1,169 participants from the Alzheimer’s Disease Neuroimaging Initiative (ADNI). Participants had in vivo measures of LB pathology (assessed using an alpha-synuclein seed amplification assay), amyloid-beta (Aβ) and phosphorylated tau (p-tau) levels in cerebrospinal fluid (CSF), and neuropsychiatric symptoms evaluated using the Neuropsychiatric Inventory-Questionnaire (NPI-Q). Logistic and Cox proportional hazards regression models were used to assess cross-sectional and longitudinal effects, respectively, adjusting for age, sex, and cognitive status. Participants had a mean baseline age of 73.05 (SD 7.22) years, 47.13% were women, 426 (36.44%) cognitively unimpaired, and 743 (63.56%) cognitively impaired. In cross-sectional analyses, LB pathology was associated with higher rates of anxiety, apathy, motor disturbances, and appetite disturbances. In longitudinal analyses, LB pathology increased the risk of developing psychosis and anxiety. These effects were independent of Aβ and p-tau. Our results suggest that in vivo-measured LB pathology is closely associated with neuropsychiatric symptoms across the AD continuum. These findings underscore the potential of in vivo LB detection as a marker for identifying individuals at increased risk of neuropsychiatric symptoms, both in clinical trials and in clinical practice.

## Introduction

Co-pathology is frequently observed in individuals with Alzheimer’s disease (AD) dementia, with alpha-synuclein deposition being one of the most common findings (1, 2). Intracellular aggregates of alpha-synuclein are denominated Lewy bodies (LB) and are the hallmark of Lewy body dementia (LBD). LBD is clinically characterized by progressive cognitive impairment, parkinsonism, and rapid eye movement (REM) sleep behavior disorder, and is commonly associated with prominent neuropsychiatric symptoms (3). Post-mortem studies report that 33–66% of individuals with AD exhibit abnormal brain alpha-synuclein aggregates (4), which have been shown to exacerbate cognitive decline (5) and brain hypometabolism (6) in living humans. Therefore, a deeper understanding of the impact of LB co-pathology in AD is essential to more accurately characterize the clinical presentations and progression of the disease.

Neuropsychiatric symptoms are common and debilitating clinical manifestations in AD, affecting 60–90% of individuals with this condition (7–10). The prevalence of neuropsychiatric symptoms increases as AD pathology becomes more severe (11), and a wide body of literature demonstrates their association with greater functional impairment (12–14). Post-mortem studies support a higher frequency of neuropsychiatric symptoms in individuals with AD and LB co-pathology. For example, Chung, Babulal (15) found higher rates of delusions, hallucinations, and aberrant motor behavior in individuals with AD and LB when compared to AD without LB. Other authors observed a higher prevalence of hallucinations, anxiety, irritability, nighttime behaviors, appetite disturbances, agitation, and apathy in individuals with concomitant AD and LB pathology (16–18). To date, however, the effects of LB pathology measured in vivo on neuropsychiatric symptoms in AD remain underexplored.

Recent advances in alpha-synuclein seed amplification assays (SAAs) have enabled accurate detection of in vivo LB pathology that is comparable to the gold standard post-mortem confirmation (4, 19). This technique provides an opportunity to study the effects of LB in living individuals, allowing earlier identification of pathology and facilitating longitudinal tracking of disease progression, thus addressing some of the limitations inherent in retrospective post-mortem analyses. In light of these advancements, the present study was designed to evaluate the cross-sectional and longitudinal effects of in vivo-detected LB pathology on distinct neuropsychiatric symptoms in a large sample of cognitively unimpaired (CU) and cognitively impaired (CI) individuals across the AD continuum.

## Materials and methods

### Participants

We used data from the Alzheimer’s Disease Neuroimaging Initiative (ADNI), a longitudinal, multicenter study designed to develop clinical, imaging, genetic, and biochemical biomarkers for the early detection and tracking of AD (http://adni.loni.usc.edu). ADNI’s inclusion criteria included having a study partner with frequent contact with the participant, age between 55 and 90 years, a Geriatric Depression Scale (GDS) score less than 6, and a Modified Hachinski Ischemic Score less than or equal to 4. CU individuals had a Mini-Mental State Exam (MMSE) score between 24 and 30 and a Clinical Dementia Rating global score (CDR-GS) of 0. Individuals with mild cognitive impairment (MCI) had a MMSE score between 24 and 30 and a CDR-GS of 0.5, with a Memory Box score of at least 0.5. Participants with AD dementia had an MMSE score between 20 and 24 and a CDR-GS of 0.5 or 1. The full inclusion and exclusion criteria for ADNI can be found elsewhere (20).

For this study, we included CU and CI (comprising MCI and AD dementia) individuals from the ADNI cohort, for whom alpha-synuclein status was determined using the cerebrospinal fluid (CSF) alpha-synuclein SAA test. Participants were also required to have a clinical and neuropsychiatric assessment within two years of the alpha synuclein SAA status. Their first neuropsychiatric assessment was defined as their baseline. Complete data (including clinical and neuropsychiatric assessments) were available for 1,169 individuals, and 977 participants had CSF amyloid (Aβ) and tau pathology measurements within two years of the baseline assessment. For the longitudinal analyses, we analyzed data from individuals who had at least two neuropsychiatric assessments within a 10-year period after their baseline. All data were downloaded from the ADNI data repository in July 2024. Institutional Review Boards of all participating sites approved the ADNI study, and all research participants or their authorized representatives provided written informed consent.

### Neuropsychiatric symptoms

Neuropsychiatric symptoms were assessed using the Neuropsychiatric Inventory-Questionnaire (NPI-Q) (21). The NPI-Q is a validated, self-administered questionnaire completed by informants covering the following neuropsychiatric domains: delusions, hallucinations, agitation/aggression, dysphoria/depression, anxiety, euphoria/elation, apathy/indifference, disinhibition, irritability/lability, motor disturbances, nighttime behavioral disturbances, and appetite/eating disturbances. Symptoms are rated for severity on a three-point scale (1 = mild, 2 = moderate, 3 = severe). In ADNI, the NPI-Q was administered at baseline and every six months for two years, then annually thereafter. For this study, individuals were categorized as positive (NPI-Q score of 1, 2, or 3) or negative (NPI-Q score of 0) for each core neuropsychiatric domain. Delusions and hallucinations were combined as “psychosis” for our analyses, due to their low baseline prevalence in our sample.

### CSF biomarkers

The presence of alpha-synuclein aggregates (LB pathology) was detected using an SAA performed at the Amprion Clinical Laboratory (22). CSF samples were classified as alpha-synuclein aggregates “detected”, “not detected”, or “indeterminate” (indicating that a result determination could not be made for a sample after being tested twice). A total of nine samples were classified as “indeterminate” and excluded from the analyses (Supplementary Fig. 1). CSF Aβ 1–42 (Aβ1–42) and tau phosphorylated at threonine 181 (p-tau181) were quantified using fully automated Elecsys immunoassays (Roche Diagnostics). Measurements outside the analytical range (< 200 pg/mL or > 1700 pg/mL for Aβ1–42; <8 pg/mL or > 120pg/mL for p-tau181) were set to the lower or upper detection limit, as previously done (23). Aβ positivity was defined as CSF Aβ1–42 < 976.6 pg/mL, and tau positivity as p-tau181 > 24 pg/mL, as previously described (24).

#### Statistical analysis

Differences in the rate of neuropsychiatric symptoms (categorized as present or absent) at baseline between LB− (samples with no detected α-syn aggregates) and LB+ (samples with detected α-syn aggregates) individuals were investigated using logistic regression analyses adjusting for age, sex, and cognitive status (model 1, Supplementary Material), with results reported as odds ratio (OR) and 95% confidence interval (CI). Next, in baseline cross-sectional analyses, we explored the effects of Aβ, p-tau and LB pathologies on neuropsychiatric symptoms. All three pathologies were used in the same model alongside sex, age, and cognitive status (model 2, Supplementary Material). To decrease the risk of false positive results, model 2 was performed only for neuropsychiatric symptoms showing significant statistical association with LB in model 1. Dichotomized Aβ and p-tau scores, as described above, were used to facilitate the comparison of estimates. After identifying pathologies with significant independent effects on neuropsychiatric symptoms in model 2, we performed post-hoc analyses to test the interaction between them. Additionally, as a sensitivity analysis, we replicated model 1 stratifying the population into CU and CI groups.

We performed longitudinal analyses to investigate the effects of LB on the development of neuropsychiatric symptoms. Only individuals who did not exhibit each specific symptom at baseline were included in these analyses, and the event was defined as the first onset of that symptom during follow-up. To compare the time-to-event data between groups (individuals with or without LB pathology), Kaplan-Meier survival curves were generated. Additionally, we used Cox proportional hazards regression models to account for sex, age, and cognitive status (model 3, Supplementary Material), with results reported as hazard ratios (HR) and 95% CI. Next, Aβ, p-tau and LB pathologies were included in the same Cox proportional hazard regression model together with sex, age, and cognitive status (model 4, Supplementary Material). Model 4 was performed only for neuropsychiatric symptoms showing significant statistical association with LB in model 3. After identifying pathologies with significant independent effects on the development of neuropsychiatric symptoms in model 4, we performed post-hoc analyses to test the interaction between them. Additionally, as a sensitivity analysis, we replicated model 3 stratifying the population into CU and CI groups. A two-sided p-value of less than 0.05 was considered statistically significant. Multiple comparison corrections were performed using the false discovery rate method (25) at alpha = 0.05, applying correction per model (models 1 and 3 with 11 analyses each, models 2 and 4 with 3 analyses for each neuropsychiatric domain). Finally, to assess model adequacy, we performed standard diagnostic analyses, including the Hosmer-Lemeshow goodness-of-fit test and link tests for logistic regressions, as well as Schoenfeld residuals tests to evaluate the proportional hazards assumption in Cox models (for details, see Supplementary Material, Supplementary Tables 1 and 2). All analyses were conducted using Stata version 18.0 (StataCorp, College Station, Texas, USA).

## Results

We analyzed data from 1,169 participants, of whom 977 had CSF Aβ and p-tau measurements. The flowchart is presented in Supplementary Fig. 1. The mean (SD) age at baseline was 73.05 (7.22) years, and 551 (47.13%) were women. A total of 426 (36.44%) individuals were classified as CU and 743 (63.56%) as CI at baseline. Baseline demographic and clinical characteristics are shown in Table 1.

A total of 850 participants (72.71%) had two or more neuropsychiatric assessments using the NPI-Q within 10 years from baseline. For those participants, the mean and median observation times were 2.64 and 1.99 years, respectively (SD = 2.13; interquartile range = 1.03–3.02; with maximum and minimum follow-up times of 9.9 and 0.45 years, respectively). Participants had a mean and median number of observations of 3.46 and 3, respectively (SD = 1.70; interquartile range = 2–4; maximum of 11 and minimum of 2 observations, respectively).

### Associations between LB pathology and baseline neuropsychiatric symptoms

**Figure 1:**
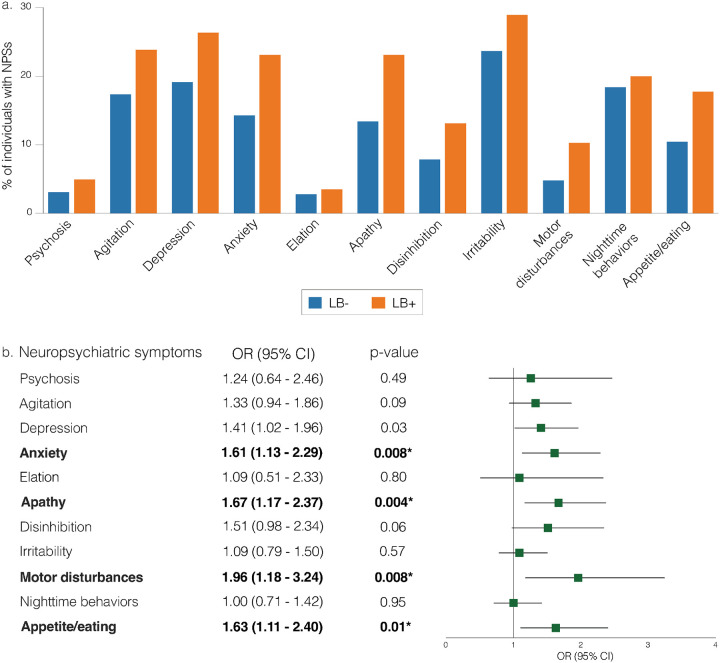
Cross-sectional associations between LB and neuropsychiatric symptoms. Figure 1a shows the percentage of individuals with positive neuropsychiatric symptoms in each NPI-Q domain. LB− and LB+ represent participants with CSF samples classified as α-synuclein aggregates “not detected” and “detected”, respectively. Figure 1b shows the ORs and 95% CIs from logistic regression models investigating differences in the rate of neuropsychiatric symptoms between LB− and LB+ individuals (OR>1 indicates higher rates of neuropsychiatric symptoms in LB+ compared to LB-). Models were adjusted for age, sex, and cognitive status (model 1, Supplementary Material). P-values in bold are statistically significant after correcting for multiple comparisons using the false discovery rate method at alpha = 0.05. **Abbreviations:** LB, Lewy body; CSF, cerebrospinal fluid; NPI-Q, Neuropsychiatric Inventory Questionnaire; OR, odds ratio; CI, confidence interval.

The frequency of neuropsychiatric symptoms at baseline is presented in Fig. 1a. In cross-sectional analyses, LB + individuals showed higher rates of anxiety (OR = 1.61, 95% CI = 1.13 to 2.29, p-value = 0.008), apathy (OR = 1.67, 95% CI = 1.17 to 2.37, p-value = 0.004), motor disturbances (OR = 1.96, 95% CI = 1.18 to 3.24, p-value = 0.008), and appetite disturbances (OR = 1.63, 95% CI = 1.11 to 2.40, p-value = 0.01) compared to LB− individuals (Fig. 1b, model 1). No significant differences were observed for psychosis, agitation, depression, elation, disinhibition, irritability, or sleep (Fig. 1b, model 1) after controlling for multiple comparisons.

**Figure 2:**
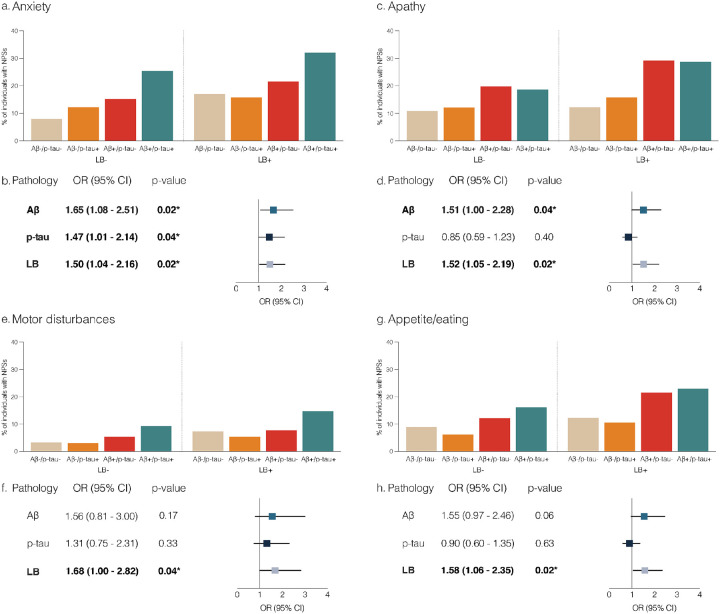
Effects of LB, Aβ, and p-tau on neuropsychiatric symptoms at baseline. Figures 2a, c, e, and g show the percentage of individuals with positive anxiety, apathy, motor disturbances, and appetite/eating disturbances, respectively, according to the NPI-Q and categorized by Aβ, p-tau, and LB status. We selected these domains based on their significant statistical association with LB in model 1. Figures 2b, d, f, and h show the ORs and 95% CIs from logistic regression models exploring the effects of Aβ, p-tau and LB pathologies on neuropsychiatric symptoms. All three pathologies were included in the same model, along with sex, age, and cognitive status (model 2, Supplementary Material). OR>1 indicates higher rates of neuropsychiatric symptoms in the presence of the pathology. P-values in bold are statistically significant after correcting for multiple comparisons within each neuropsychiatric domain using the false discovery rate method at alpha = 0.05. Abbreviations: LB, Lewy body; Aβ, amyloid-beta; NPI-Q, Neuropsychiatric Inventory Questionnaire; OR, odds ratio; CI, confidence interval.

Figures 2a, c, e, and g show the percentage of individuals presenting with anxiety, apathy, motor disturbances, or appetite/eating disturbances (statistically significant in model 1) according to their Aβ, p-tau, and LB pathology status at baseline. When including all three pathologies in the model (model 2), the presence of Aβ, p-tau, and LB was associated with higher rates of anxiety (Fig. 2b). For apathy, the presence of Aβ and LB was associated with a higher prevalence of symptoms (Fig. 2d). For motor disturbances and appetite disturbances, only LB was associated with a higher prevalence of symptoms (Fig. 2f, h).

The prevalence of neuropsychiatric symptoms according to AD and LB pathology can be found in Supplementary Table 3. We found no significant interactions between LB and Aβ on anxiety (OR = 0.69, 95% CI = 0.28 to 1.72, p-value = 0.43) or apathy (OR = 1.48, 95% CI = 0.57 to 3.81, p-value = 0.41).

Similarly, we observed no interaction between LB and p-tau on anxiety (OR = 0.77, 95% CI = 0.36 to 1.64, p-value = 0.50). When stratifying participants by cognitive status, we found no differences in the frequency of neuropsychiatric symptoms in CU participants (Supplementary Table 4). In CI individuals, the presence of LB was associated with higher rates of anxiety, apathy, disinhibition, motor disturbances, and appetite/eating disturbances (Supplementary Table 4).

### Associations between LB pathology and longitudinal neuropsychiatric symptoms

**Figure 3:**
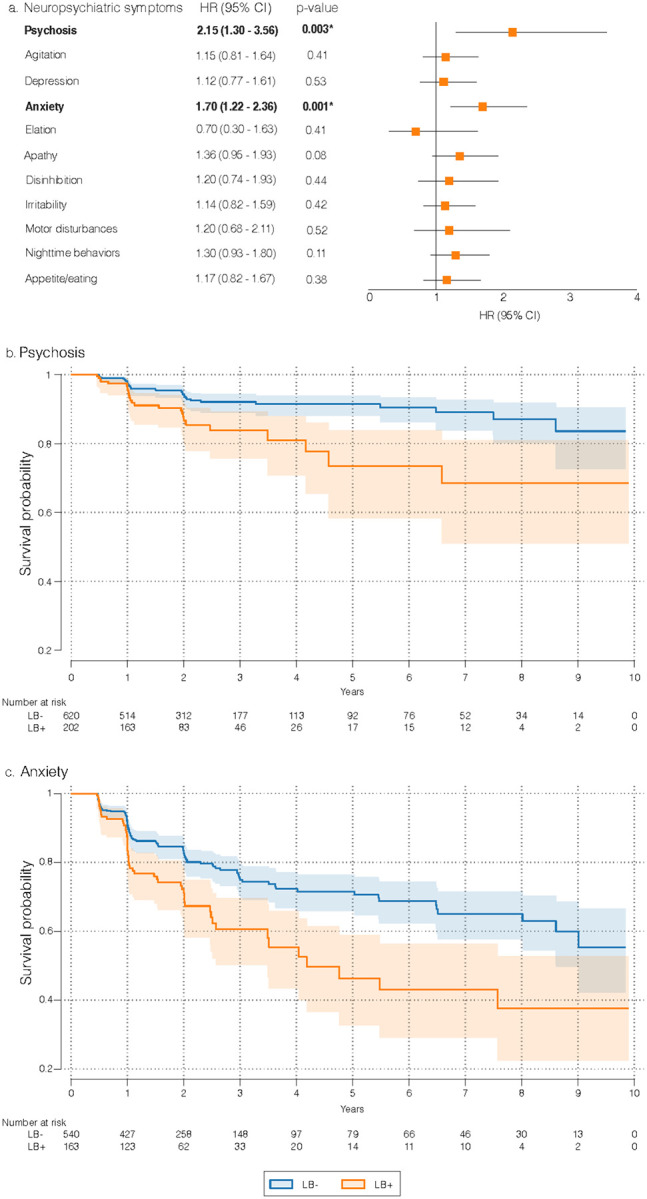
Effects of LB on the development of neuropsychiatric symptoms. Figure 3a shows the HR and 95% CIs from Cox proportional hazard regression models investigating the effects of LB on the development of neuropsychiatric symptoms, adjusted for sex, age, and cognitive status (model 3, Supplementary Material). P-values in bold are statistically significant after correcting for multiple comparisons using the false discovery rate method at alpha = 0.05. Figures 3b and c show the Kaplan-Meier survival curves, with shaded areas indicating the 95% CI. Abbreviations: LB, Lewy body; HR, hazard ratio; CI, confidence interval.

Cox proportional hazards regression models showed that LB + individuals had a higher risk of developing psychosis (HR = 2.15, 95% CI = 1.30 to 3.56, p-value = 0.003) and anxiety (HR = 1.70, 95% CI = 1.22 to 2.36, p-value = 0.001) during follow-up compared to LB− individuals (model 3, Fig. 3a). The Kaplan-Meier survival curves illustrating time to onset of psychosis and anxiety symptoms are presented in Figs. 3b and 3c. No differences in risk were observed for agitation, depression, elation, apathy, disinhibition, irritability, motor disturbances, sleep, or appetite disturbances (model 3, Fig. 3a). After including Aβ and p-tau in the model, the presence of Aβ and LB was associated with higher risks of developing psychosis and anxiety during follow-up (model 4, Table 2).

We found no significant interactions between LB and Aβ for psychosis (HR = 0.83, 95% CI = 0.15 to 5.51, p-value = 0.83) or anxiety (HR = 0.99, 95% CI = 0.44 to 2.21, p-value = 0.98). When stratifying participants by cognitive status, Cox proportional hazard regression models showed no differences in the risk of developing neuropsychiatric symptoms in CU individuals when comparing LB + and LB− (Supplementary Table 5). For CI individuals, the presence of LB was associated with a higher risk of developing psychosis and anxiety (Supplementary Table 5).

## Discussion

This study aimed to explore the cross-sectional and longitudinal effects of LB pathology on neuropsychiatric symptoms across the AD continuum. We observed that in vivo presence of LB was associated with higher baseline rates of anxiety, apathy, motor disturbances, and appetite disturbances. Furthermore, in longitudinal analyses, the presence of LB was associated with an increased risk of developing psychotic and anxiety symptoms. The effects of LB were independent of Aβ and p-tau pathology and were more pronounced in CI individuals. These findings reinforce that LB pathology contributes to the development of neuropsychiatric symptoms in AD and suggest that in vivo detection of LB can assist in identifying individuals at risk for psychosis and anxiety.

Our findings are consistent with previous research demonstrating high rates of behavioral disturbances in patients presenting LB pathology (26). Specifically, results from our cross-sectional analyses provide new evidence that LB pathology detected in vivo is associated with increased rates of anxiety, apathy, motor disturbances, and appetite/eating disturbances independent of Aβ or p-tau pathology. These symptoms have been previously shown to occur frequently in individuals with a clinical diagnosis of LBD, with reported rates ranging from 40–60% for anxiety (27–31), 30–55% for apathy (29–31), 20–60% for motor disturbances (30, 31), and 15–30% for appetite/eating disturbances (29, 31). The spectrum of neuropsychiatric disturbances observed in individuals with a clinical diagnosis of LBD is broad, and includes visual hallucinations, delusions, REM sleep behavior disorder, and depression (26). In our study, we observed a trend toward a higher frequency of agitation, depression, and disinhibition that did not reach statistical significance after adjusting for covariates and multiple comparisons. One possible explanation for the weak association of these symptoms in our analysis is that we studied participants at earlier stages of LB pathology compared with those who reach full criteria for a clinical diagnosis of LBD. This observation aligns with literature suggesting that in vivo detection of LB can identify individuals in the early stages of symptom development (32). In this sense, we propose that anxiety, apathy, motor disturbances, and appetite/eating disturbances are early neuropsychiatric manifestations potentially arising from LB pathology across the AD continuum.

Our findings suggest that LB and AD pathology (Aβ and p-tau) independently contribute to elevated baseline rates of anxiety, while LB and Aβ pathology significantly increase the risk of developing anxiety over a 10-year period. Clinical and preclinical evidence suggests that LB pathology accumulation in the limbic system plays a role in anxiety development in LBD (28). However, most of the supporting evidence thus far comes from post-mortem studies, which have demonstrated that LB and AD pathology independently contribute to elevated rates of anxiety (16, 18). When comparing individuals with both AD and LB pathology to those with AD alone, findings have been mixed. One study reported that anxiety was more frequent in individuals with LB and AD pathology (17), while others did not observe this association (15, 16, 18). In contrast, our current study employs in vivo detection of LB pathology, which allows us to examine the risk of developing anxiety over a 10-year period. In line with our observations showing a higher risk of developing anxiety in participants with Aβ pathology, previous studies have found that CU individuals with high Aβ burden experience increasing anxiety levels over time (33, 34). Collectively, our findings reinforce the notion that LB and AD pathology independently contribute to anxiety symptoms across the AD continuum and highlight the potential of in vivo LB detection as a valuable marker for identifying individuals at risk for developing high levels of anxiety.

While delusions and hallucinations are hallmark neuropsychiatric symptoms in diagnosed LBD, we did not find differences in psychosis rates in our cross-sectional analyses. On the other hand, over a 10-year follow-up period, we found that individuals with LB pathology were more likely to develop psychotic symptoms. The association between LB pathology and psychotic symptoms has been well-established in post-mortem studies (15–18), and the presence of neocortical LB has been linked to psychosis in LBD (35). Unlike our cross-sectional findings, Quadalti, Palmqvist (5) observed higher rates of hallucinations in CI individuals with in vivo detected LB pathology in cross-sectional analyses. This discrepancy may derive from the low baseline rates of psychotic symptoms, including hallucinations and delusions, in our study population, which might have limited our power to detect cross-sectional differences. This can potentially be attributed to self-selection bias, as individuals with high levels of psychosis are less likely to enroll in AD-related studies. Overall, our findings support an association between LB pathology measured in vivo and the development of psychotic symptoms during the AD continuum.

Findings from this study should be interpreted with consideration of the following limitations. The cohort consisted of individuals motivated to participate in a dementia study, which may introduce self-selection bias and limit the generalizability of our findings to the broader elderly population. The NPI-Q provides a brief assessment of neuropsychiatric symptoms and may not capture the full complexity and nuance of psychiatric symptomatology. For instance, it may not detect specific nighttime behaviors typically seen in REM sleep behavior disorder. As the NPI-Q is completed by informants, recall bias may influence the accuracy of reported symptoms, particularly for symptoms with subtle or fluctuating presentations. Varying follow-up durations and assessment among participants may introduce bias in the longitudinal analyses. To address this, we employed survival analyses to account for censored data, though residual bias cannot be entirely ruled out. It would be desirable to replicate our results in a population-based cohort.

To conclude, this study demonstrates that LB pathology measured in vivo is associated with a higher frequency of neuropsychiatric symptoms. These effects were independent of Aβ and p-tau pathologies. Our findings highlight the value of in vivo LB detection in the identification of individuals at a high risk for anxiety and psychosis. Future studies with expanded longitudinal follow-up and comprehensive neuropsychiatric assessments will be critical in further elucidating the temporal relationship between in vivo-measured LB pathology and neuropsychiatric symptom development.

## Figures and Tables

**Table 1:** Baseline demographic and clinical characteristics.

	Overall (*N*=1,169)	LB− (*N*=888)	LB+ (*N*=281)
Age, y, mean (SD)	73.05 (7.22)	72.59 (7.18)	74.49 (7.16)
Sex, No. (%)			
Female	551 (47.13)	437 (49.21)	114 (40.57)
Male	618 (52.87)	451 (50.79)	167 (59.43)
Race, No. (%)			
White	1091 (93.33)	821 (92.45)	270 (96.09)
Black	41 (3.51)	36 (4.05)	5 (1.78)
Asian	20 (1.71)	15 (1.69)	5 (1.78)
Hawaiian/Other PI	1 (0.09)	1 (0.11)	0
More than one	12 (1.03)	11 (1.24)	1 (0.36)
Unknown	2 (0.17)	2 (0.23)	
Ethnicity, No. (%)			
Not Hispanic/Latino	1127 (96.41)	853 (96.06)	274 (97.51)
Hispanic/Latino	36 (3.08)	30 (3.38)	6 (2.14)
Unknown	6 (0.51)	5 (0.56)	1 (0.36)
Cognitive status, No. (%)			
CU	426 (36.44)	347 (39.08)	79 (28.11)
CI	743 (63.56)	541 (60.92)	202 (71.89)
MMSE, mean (SD)	1168 (27.02)	27.35 (2.85)	25.95 (3.75)
CDR-SB, mean (SD)	1.74 (2.14)	1.55 (2.05)	2.34 (2.30)
APOE ε4, No. carries (%)	530 (46.61)	388 (44.04)	142 (50.53)
CSF Aβ1–42, No. positive (%)	604 (61.82)	417 (57.12)	187 (75.71)
CSF Aβ1–42, pg/mL, mean (SD)	934.51 (446.97)	980.31 (448.36)	799.18 (415.09)
CSF p-tau181, No. positive (%)	525 (53.74)	384 (52.60)	141 (57.09)
CSF p-tau181, pg/mL, mean (SD)*	28.65 (15.30)	28.66 (15.85)	28.62 (13.58)
Participants with follow-up data, No. (%)	850 (72.71)	638 (71.84)	212 (75.44)
Follow-up, y, mean (SD)*	2.64 (2.13)	2.75 (2.21)	2.32 (1.82)

Table 1 presents baseline demographic and clinical characteristics of the overall study population, stratified by LB status (LB− and LB+).

Missing CSF Aβ1–42 and p-tau181: 158 LB−, 34 LB+. Missing MMSE: 1 LB−. Missing CDR-SB:. 2 LB−, 1 LB+. Missing APOE ε4: 7 LB−

* For participants with available follow-up data.

Abbreviations: LB, Lewy body; SD, standard deviation; y, years; PI, Pacific Islander; CU, cognitively unimpaired; CI, cognitively impaired; CSF, cerebrospinal fluid.

**Table 2:** Effects of LB, Aβ, and p-tau on the development of neuropsychiatric symptoms.

	Aβ		p-tau		LB	
	HR (95% CI)	p-value	HR (95% CI)	p-value	HR (95% CI)	p-value
Psychosis	3.40 (1.54 to 7.49)	0.002	1.78 (0.98 to 3.22)	0.05	1.81 (1.08 to 3.04)	0.02
Anxiety	1.85 (1.26 to 2.71)	0.002	1.03 (0.73 to 1.46)	0.82	1.61 (1.15 to 2.24)	0.005

Table 2 presents the results of longitudinal analyses using Cox proportional hazards regression exploring the effects of Aβ, p-tau and LB status on the development of neuropsychiatric symptoms. All three pathologies were included in the same model, alongside sex, age, and cognitive status (model 4, Supplementary Material). For each neuropsychiatric symptom (psychosis and anxiety), HR with 95% CI and corresponding p-values are provided. HR > 1 indicates an increased risk of developing the symptom in the presence of the pathology.

Abbreviations: Aβ, amyloid-beta; LB, Lewy body; HR, hazard ratio; CI, confidence interval.
